# The effects of Jiao-Tai-Wan on sleep, inflammation and insulin resistance in obesity-resistant rats with chronic partial sleep deprivation

**DOI:** 10.1186/s12906-017-1648-9

**Published:** 2017-03-23

**Authors:** Xin Zou, Wenya Huang, Fuer Lu, Ke Fang, Dingkun Wang, Shuyong Zhao, Jiming Jia, Lijun Xu, Kaifu Wang, Nan Wang, Hui Dong

**Affiliations:** 1 Institute of Integrated Traditional Chinese and Western Medicine, Tongji Hospital, Tongji Medical College, Huazhong University of Science and Technology, Wuhan, 430030 People’s Republic of China; 2 Shijiazhuang YiLing Pharmaceutical Co., Ltd., Shijiazhuang, 050035 People’s Republic of China; 3 Department of Radiology, Tongji Hospital, Tongji Medical College, Huazhong University of Science and Technology, Wuhan, 430030 People’s Republic of China

**Keywords:** Jiao-Tai-Wan, Sleep deprivation, Inflammation, Insulin resistance, Circadian clock

## Abstract

**Background:**

Jiao-Tai-Wan (JTW), composed of *Rhizome Coptidis* and *Cortex Cinnamomi*, is a classical traditional Chinese prescription for treating insomnia. Several in vivo studies have concluded that JTW could exert its therapeutical effect in insomnia rats. However, the specific mechanism is still unclear. The present study aimed to explore the effect of JTW on sleep in obesity-resistant (OR) rats with chronic partial sleep deprivation (PSD) and to clarify its possible mechanism.

**Methods:**

JTW was prepared and the main components contained in the granules were identified by 3D-High Performance Liquid Chromatography (3D-HPLC) assay. The Male Sprague-Dawley (SD) rats underwent 4 h PSD by environmental noise and the treatment with low and high doses of JTW orally for 4 weeks, respectively. Then sleep structure was analyzed by electroencephalographic (EEG). Inflammation markers including high-sensitivity C reactive protein (hs-CRP), tumor necrosis factor-α (TNF-α) and interleukin-6 (IL-6) levels were examined in the rat plasma. Meanwhile, metabolic parameters as body weight increase rate, fasting plasma glucose (FPG), fasting insulin (FINS) levels and insulin resistance index (HOMA-IR) were measured. The expressions of clock gene cryptochromes (Cry1 and Cry2) and inflammation gene nuclear factor-κB (NF-κB) in peripheral blood monocyte cells (PBMC) were also determined.

**Results:**

The result showed that the administration of JTW significantly increased total sleep time and total slow wave sleep (SWS) time in OR rats with PSD. Furthermore, the treatment with JTW reversed the increase in the markers of systemic inflammation and insulin resistance caused by sleep loss. These changes were also associated with the up-regulation of Cry1 mRNA and Cry 2 mRNA and the down-regulation of NF-κB mRNA expression in PBMC.

**Conclusions:**

This study suggests that JTW has the beneficial effects of improving sleep, inflammation and insulin sensitivity. The mechanism appears to be related to the modulation of circadian clock and inflammation genes expressions in PBMC.

## Background

Sleep loss has become a global problem due to social-economic factors in the modern society. Since sleep is important to recover our energy, inadequate sleep is always thought to be a reason for weight loss [1]. However, growing evidence suggests that sleep curtailment is a new risk factor for the development of obesity and type 2 diabetes [2, 3]. For instance, in the United States, self-reported sleep duration has decreased by 1.5 ~ 2 h in the last 50 years coinciding with an increase in obesity and diabetes [4]. In China, nearly one-third of the population reported poor sleep quality or shortened sleep duration (≤6 h). Compared to subjects with good quality sleep who slept for 6-8 h per night, those who have poor sleep quality and short sleep duration were more likely to have diabetes [5].

With regard to the pathophysiology of sleep loss-associated obesity and diabetes, present literature highlights the role of inflammation and insulin resistance (IR) after sleep curtailment [6]. Clinical studies have found that plasma inflammatory cytokines increase significantly with the activation of cellular inflammation [7]. These alterations could be observed even after one night of sleep deprivation [8]. The existence of chronic low-grade inflammation may be associated with IR and precedes the onset of type 2 diabetes in adults [9]. Thus getting enough good sleep plays an important role of maintaining metabolic health. In addition, insomnic patients often take sedative-hypnotics, antipsychotics, antidepressants and anti-anxiety medications. Abnormal long sleep duration and increased appetite associated with these drugs may also cause insulin resistance and high risk of weight-gain. Therefore, the therapy with a dual-effect of sleep and IR improvement is promising [10].

Jiao-Tai-Wan (JTW), composed of *Rhizome Coptidis* (*Coptis chinensis Franch*, *Ranunculaceae*) and *Cortex Cinnamomi* (*Cinnamomum cassiaPresl*, *Lauraceae*), is a classical traditional Chinese prescription for treating insomnia [11]. The application of JTW to treat insomnia can be traced back to Han Shi Yi Tong in Ming Dynasty. The function of *Rhizome Coptidis* (Huanglian in Chinese) is to clear away heart fire and the function of *Cortex Cinnamomi* (Rougui in Chinese) is to warm kidney water. According to a survey, *Rhizome Coptidis* was one of the top 10 individual Chinese herbs prescribed for insomnia in Taiwan during 2002 [12]. In a meta-analysis within 217 reviewed studies, JTW was one of the 10 most frequently examined standardized Chinese herbal formulas for insomnia [13]. In in vivo studies, JTW has been proven to play its sedative and hypnotic role on insomnia rats [14, 15]. Previous researches have demonstrated that JTW also exhibits hypoglycemic effect and insulin sensitizing activity in diabetic rodents and diabetic patients [16, 17]. The possible mechanisms may be related to suppressing gluconeogenesis by activating adenosine 5′-monophosphate-activated protein kinase in the liver and enhancing insulin signaling through phosphatidylinositol 3-kinase pathway in the skeletal muscle [18, 19]. Given that sleep loss induces inflammation and IR, we hypothesize that JTW may have the potential to alleviate inflammation and improve insulin sensitivity after sleep curtailment. Also, one study suggested that multiple-dose oral administration of JTW may improve its absorption in insomnic rats compared with normal rats, which will increase the bioavailability, and give full play of its therapeutical effect [20]. Therefore, we established a rat model of chronic partial sleep deprivation (PSD) and investigated the effects and mechanisms of JTW on sleep structure, inflammation and IR.

## Methods

### Animals

Male Sprague-Dawley (SD) rats (*n* = 250, 10 weeks, 200 ± 20 g) were supplied by Hubei Province Center for Disease Control and Prevention (Wuhan, China). The rats were maintained on 12 h light-12 h dark cycle in a humidity- and temperature-controlled environment. All procedures were approved by Huazhong University of Science and Technology Ethics Committee for the use of experimental animals (IRB ID: TJ-A20131218). After fed with food and water ad libitum for 1 week, all rats were switched to a high-fat, high-energy diet (8% corn oil, 44% sweetened condensed milk, 48% standard rat chow) for 2 weeks. Then 100 rats with the lowest weight gain (about 40%) were classified as obesity-resistant (OR) rats [21]. As has been previously proved, better sleep quality may confer obesity protection in OR rats [22]. However, chronic partial sleep deprivation increases body weight even in OR rats which is mostly consistent with human conditions [23]. Accordingly, OR rat is an excellent animal model to study sleep loss-associated obesity and IR [24].

### Preparation of JTW

JTW is composed of *Rhizome Coptidis* and *Cortex Cinnamomi*. The ratio of these two herbs is 10:1 (w/w). In the present study, we use *Rhizome Coptidis* and *Cortex Cinnamomi* concentrated granules (purchased from China Resources Sanjiu Medical and Pharmaceutical Co., Ltd) for rat administration. *Rhizome Coptidis* and *Cortex Cinnamomi* were all purchased from Rui Sheng Yuan Biotechnology Co., Ltd. (Chengdu, China) and identified by Department of Chinese Medicine Authentication of Jin Chan Pharmaceutical Co., Ltd. (Hefei, China). All the samples are deposited in the herbarium of the same department with voucher number No. 472 for *Rhizoma Coptidis*, No. 353 for *Cortex Cinnamomi*. The herbs were extracted by using a method of simulated family decoction with boiling water. Then the extracts were concentrated, isolated, dried to form granules. This process of production was performed according to Good Manufacturing Practice (GMP) for Drugs (Chinese FDA, 2010 Version) to ensure the quality control. And the obtained products were re-identified by Guangdong Institute for Food and Drug Control (Guangzhou, China). As a result, 0.5 g of *Rhizome Coptidis* granule efficacy is equivalent to 3 g decoction pieces and 1 g of *Cortex Cinnamomi* granule efficacy is equivalent to 3 g decoction pieces. We mixed the granules together by distilled warm water and administered to the rat at the decoction (not granule) pieces dosage of 1.1 g/kg and 2.2 g/kg body weight/day, respectively. The calculation method was as follows: for a person with body weight of 60 kg, the high dose of daily JTW intake is 22 g (20 g of *Rhizoma Coptidis* and 2 g of *Cortex Cinnamomi*). According to the conversion of human doses (Chinese Pharmacoepia, 2010) to rat equivalent doses based on body surface areas, the daily dose of JTW intake in rats is 6 times higher than human. Therefore, we chose 2.2 g/kg body weight as the daily dose by gavage to the rat.

### Determination of the main chemical constituents in JTW by 3D-HPLC analysis

**Fig. 1:**
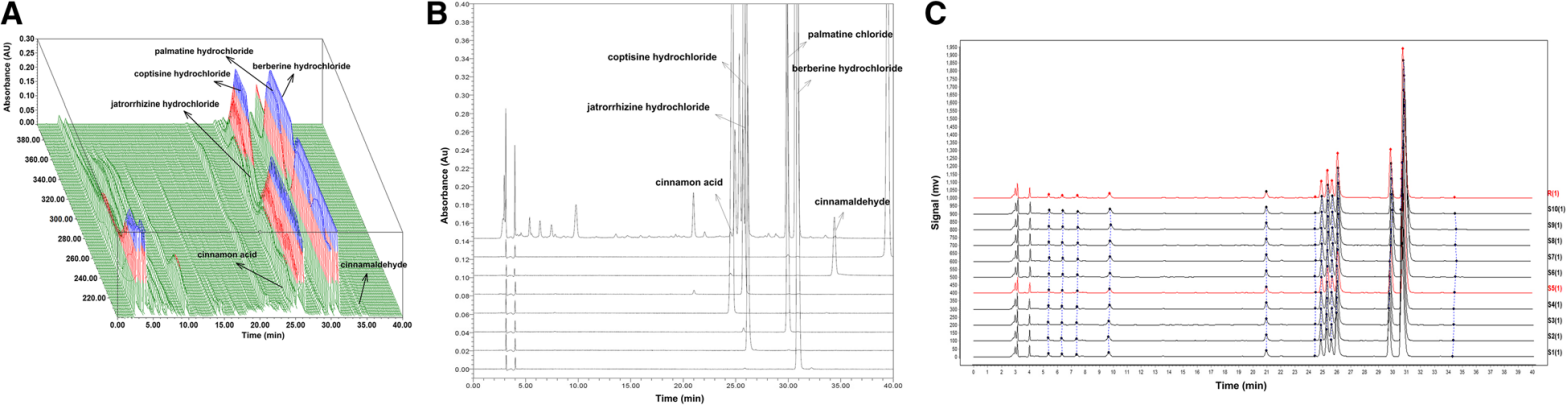
The 3D-HPLC chromatogram of JTW. **a** 3D-HPLC fingerprint of JTW; **b** HPLC chromatograms of six standard chemicals; **c** HPLC chromatogram from ten different batches of JTW to detect the similarity. Standardization of JTW was done using HPLC fingerprinting with chemical standards including cinnamon acid, jatrorrhizine hydrochloride, coptisine hydrochloride, palmatine hydrochloride, berberine hydrochloride, cinnamaldehyde

The main chemical constituents in JTW were identified by 3D-High Performance Liquid Chromatography (3D-HPLC) method (Fig. 1a). The reference standards of cinnamaldehyde, cinnamon acid, palmatine chloride and berberine hydrochloride were purchased from National Institutes for Food and Drug Control (Beijing, China). The reference standards of coptisine hydrochloride and jatrorrhizine hydrochloride were purchased from Tauto Biotechnology Ltd. (Shanghai, China) (Fig. 1b). For the analysis, the drying powder *Rhizome Coptidis* (0.25 g) and *Cortex Cinnamomi* (0.05 g) were accurately weighed, mixed and extracted with 100 ml of methanol in an ultrasonic bath for 60 min. Additional methanol was added to compensate for any lost volume. Then the resulting solution was filtered through a 0.45 μm membrane filter prior to HPLC injection. HPLC was performed on a Waters 2695-2996HPLC system (Waters Corporation, Milford, MA, USA) equipped with an Agilent TC-C18 column (4.6 mm × 250 mm, 5um). The mobile phase included acetonitrile (A) and 0.05 mol/l potassium dihydrogen phosphate (B) at a flow speed of 1.0 ml/min in the condition of column temperature 30 °C. The detection wavelength was set at 270 nm and the sample injection volume was 10 μl. The gradient elution was as follows: 0 ~ 5 min, 85% ~ 85% B, 5 ~ 40 min, 85% ~ 57% B. Chromatographic data was collected and analyzed by using Waters Empower 3 software. Moreover, ten batches of JTW which were supplied by China Resources Sanjiu Medical and Pharmaceutical Co., Ltd. were used to detect the similarity (Fig. 1c). The similarity of fingerprints was analyzed by professional software named Similarity Evaluation System for Chromatogram Fingerprint of Traditional Chinese Medicine (version of 2004A).

### Experimental design

Selected OR rats were switched to standard laboratory chow and randomly allocated to four groups, control group, model group, low dose JTW treated group (JTW1) and high dose JTW treated group (JTW2) (25 rats in each group). Rats in model group and JTW treated group were subjected to PSD during the light phase (4 h/day from 10:00 to 14:00) for 4 weeks. Sleep deprivation was achieved by exposing the animals to environmental noise, as previously described [23]. Meanwhile, rats in JTW1 and JTW2 treated group were respectively given low and high doses of JTW granules by intragastric administration once a day. Five rats in each group were sacrificed to collect blood samples at the 1st, 2nd, 3rd and 4th week. Blood was collected from aorta abdominalis for biochemical analysis and Peripheral Blood Mononuclear Cells (PBMC) isolation.

### Surgery, sleep recordings and analysis

Another five rats in each group underwent surgery 1 week prior to any PSD subjection or JTW intake, using standard procedures as bellow [25]. Under pentobarbital (50 mg/kg, intraperitoneal injection)-induced anesthesia, two stainless steel screws (2 mm long, 1.2 mm of diameter) attached to insulated wire were implanted in the skull over the frontal-parietal cortex for electroencephalographic (EEG) recordings. One of the electrodes was placed approximately 2 mm anterior and 5 mm to the right of bregma; another was placed approximately 2 mm anterior and 5 mm to the left of bregma. Both of the electrodes were attached to a miniature connector. All connectors were cemented in place with dental acrylic. Right after the surgical implantation, the rats were injected with Penicillin sodium (80,000 unit dose for each rat) for 2 days and allowed to recover for 7 days prior to the initiation of the experiments. At the end of the 4th week, EEG recordings were collected. For the purpose of habituation, the animals were connected to the recording apparatus at least 1 day before the experiments for EEG recordings. The miniature connectors were connected to a RM-6280C 8-channel physiological signal recorder (Chengdu Instrument Factory, Chengdu, China) via the signal input lines. Make sure the animal can move freely within a wide range. The signals were amplified and filtered (1 ~ 30 Hz) digitized at a speed of 200 ms/disc, and recorded using Model RM6280C physiological signal acquisition and processing system. The system was run for EEG recording continuously for 4 h (10:00–14:00). The EEG waveform files were saved and the waves ratio tables were exported for further analysis. The power spectrum densities, integrated and averaged, could be divided into four frequency areas: δ wave (0.5 ~ 4 Hz), θ wave (4 ~ 8 Hz),αwave (8 ~ 14 Hz), and β wave (14 ~ 30 Hz). According to rat EEG, each epoch was assigned to one of the following categories [26]: wakefulness (can be divided into two types of EEG: θ wave (4 ~ 8 Hz) when the rats in walking, climbing, exploring and focus behavior; irregular high-frequency and low-amplitude EEG activity when the rats licking the body and standing still), slow-wave sleep stage I (SWS_1_, high-amplitude, low-frequency (1 ~ 5 Hz) and spindle waves (8 ~ 14 Hz) synchronous pattern activity, and high-amplitude and low-frequency wave ratio less than 50% share in each of the statistical unit (30 s), eyes closed, usually lying on the animal’s side or curled up with head down), slow-wave sleep stage II (SWS_2_, same as SWS_1_, but high-amplitude and low-frequency wave ratio more than 50% share in each of the statistical unit), REM sleep (low-frequency θ wave, with no significant difference between the waking state, occasional body twitches while maintaining a recumbent sleep posture. Since wakefulness could not directly turn to the REM sleep state, there was always the presence of SWS sleep before the REM sleep, and REM sleep could be directly convert into wakefulness or SWS sleep. REM sleeping time generally sustained not more than 3 min). Each individual sleeping phase lasted at least 20 s, and each analysis unit was 30 s. The total sleep time, total time of slow wave sleep (SWS_total_), SWS_1_ and SWS_2_, and total time of REM sleep were calculated to analyze the differences among the four groups.

### Determination of plasma hs-CRP, TNF-α and IL-6 levels

Plasma high-sensitivity C reactive protein (hs-CRP), tumor necrosis factor-α (TNF-α) and interleukin-6 (IL-6) levels were determined using the ELISA kits (Boster Bio-engineering Co., Ltd., Wuhan, China), according to the manufacturer’s protocol.

### Measurement of body weight increase rate, FPG, FINS levels and HOMA-IR

The body weight increase rate (%) was calculated by the net weight increase (g) each week divided by the previous weight (g). Fasting plasma glucose (FPG) was measured by the glucose oxidase method using a commercially available kit (Mingdian Bio-engineering Co., Ltd., Shanghai, China) and fasting insulin (FINS) level was determined by ELISA method (Boatman Biotech Co., Ltd., Shanghai, China). The homeostasis model assessment index of IR (HOMA-IR) was calculated using the formula of FPG (mmol/L) × FINS (μIU/mL)/22.5.

### Assessment of clock gene Cry and inflammation gene NF-κB expression in PBMC

The PBMC in different groups was isolated by using a Ficoll density gradient separation kit (Haoyang Biological Manufacture Co., Ltd., Tianjin, China) as previously described [27]. The resulting cells were re-suspended in 1 ml Trizol (Ambion Inc., Austin, TX, USA) and stored at −80 °C. Total RNA was extracted according to the manufacturer’s instructions. The purity and concentration of the extracted RNA were measured by a Nucleic Acid/Protein Analyzer (Thermo, Rockford, USA). Then 1 μg of total RNA was reverse-transcribed using a Prime Script RT reagent Kit in a total reaction volume of 20 μl. cDNA was synthesized in a Mastercycler gradient PCR apparatus (Eppendorf Company, Hamburg, Germany). Then 2.0 μl of this cDNA was amplified in a 20 μl PCR reaction mixture containing 6.8 μl ddH2O, 0.4 μl forward primer, 0.4 μl reverse primer, 0.4 μl ROX Reference Dye (50×) and 10.0 μl SYBR premix EX Taq™ (TaKaRa Company, Dalian, China) with an Applied Biosystems StepOne Real-Time PCR System (StepOne, Foster City, USA). The reaction included 40 cycles of the following 3 stages: stage1, 95 °C for 30 s; stage2, 95 °C for 5 s,; stage 3, 60 °C for 30 s. The primer sequences were shown in Table 1. The method of 2^-ΔΔCT^was used to calculate the relative expression level of each group.

**Table 1:** Real-time PCR primer sequences for Cry1, Cry2 and NF-κB

Gene	Forward (5′ → 3′)	Reverse (5′ → 3′)
Cry1	5′-CAGCCAGCTGACGTGTTTCC-3′	5′-AATGCGCACGATGACTTCCA-3′
Cry2	5′-ACCGCCTGTGGGACTTGTA-3′	5′-TCGCCATAGGAGTTGTCCAAATA-3′
NF-κB	5′-GAGGGACGACACCTCTACACATA-3′	5′-CCCAAGAGTCGTCCAGGTCA-3′
β-actin	5′-AGCCATGTACGTAGCCATCC-3′	5′- CTCTCAGCTGTGGTGGTGAA-3′

### Statistical analysis

Data were presented as mean ± standard deviation (SD) and analyzed using SPSS 19.0 software. One-way analysis of variance (ANOVA) was used to determine the statistical significance, followed by a LSD post hoc test (for equal variances) or a Dunnett’s T3 post hoc test (for not assumed equal variances). *p* < 0.05 was considered statistically significant.

## Results

### 3D-HPLC profile of JTW

3D-HPLC chromatogram of JTW was shown in Fig. 1a. Six of the constituents in JTW were identified by comparing the retention time and peak height with reference standard (Fig.1b). The main constituents of JTW were as follows: cinnamon acid, jatrorrhizine hydrochloride, coptisine hydrochloride, palmatine chloride, berberine hydrochloride and cinnamaldehyde. HPLC chromatogram of JTW from ten batches was shown in Fig. 1c and the similarity coefficients were no less than 0.99

### The effect of JTW on sleep parameters in sleep-deprivated OR rats

As shown in Table 2, during the 4 h EEG recording condition, total sleep time and total SWS time were significantly reduced in OR rats with PSD (*p* < 0.01). Time spent in SWS_1_ and SWS_2_ were also decreased (*p* < 0.01). However, JTW1 and JTW2 treatment increased total sleep time, SWS_total_ time, especially SWS_1_ time in OR rats with PSD (*p* < 0.01). And JTW1 and JTW2 slightly increased the SWS_2_ time compared with the model group. As for REM time, there was no significant difference among these four groups (data not shown). The result suggests that JTW has a beneficial effect on extending the total sleeping time, especially SWS_1_ time in OR rats with PSD, changing the structure to a better state more suitable for sleeping.

**Table 2:** The effects of JTW on sleep structure in sleep-deprivated OR rats

Groups	Total sleep time (s)	Slow wave sleep (s)		
		SWS_total_	SWS_1_	SWS_2_
Control	14,100 ± 198	14,076 ± 231	7650 ± 1672	6426 ± 1640
Model	6492 ± 486^##^	6471 ± 485^##^	4467 ± 387^##^	2004 ± 730^##^
JTW1	10,450 ± 308^**^	10,380 ± 314^**^	7600 ± 273^**^	2780 ± 152
JTW2	12,513 ± 385^**^	12,486 ± 434^**^	9534 ± 402^**^	2952 ± 276

Data are expressed as mean ± SD (s, *n* = 5). SWS_total_: total slow wave sleep time; SWS_1:_ slow-wave sleep stage I time; SWS_2_: slow-wave sleep stage II time. ^##^ *p* < 0.01 vs. control group, **p < 0.01 vs. model group

### The effect of JTW on plasma inflammatory biomarkers in sleep-deprivated OR rats

**Fig. 2:**
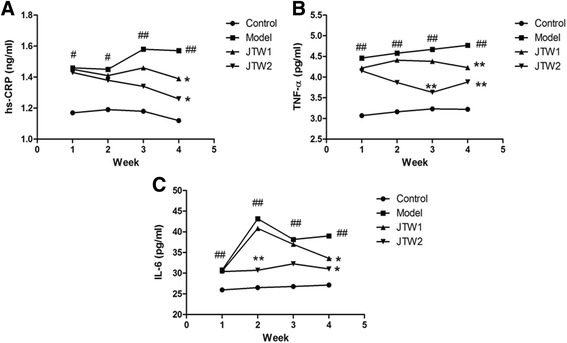
The effect of JTW on plasma inflammatory biomarkers in sleep-deprivated OR rats. JTW supplementation decreased the level of plasma inflammatory biomarkers in OR rats with PSD (**a** hs-CRP; **b** TNF-α; **c** IL-6). ^#^ *p* < 0.05, ^##^ *p* < 0.01, significantly different from control. *p < 0.05, **p < 0.01, significantly different from model group. Each point represents the mean of 5 rats

As shown in Fig. 2a-c, plasma hs-CRP, TNF-α and IL-6 levels were significantly elevated (*p* < 0.05, *p* < 0.01) each week in OR rats with PSD, indicating systemic inflammation after sleep curtailment. Treatment with JTW1 and JTW2 exhibited a decrease in the level of plasma inflammatory biomarkers compared with the model group. This reduction trend was observed throughout the deprivation period and reached statistical significance at the end of the fourth week (*p* < 0.05, *p* < 0.01). In addition, plasma TNF-α level was markedly lower (*p* < 0.05) in JTW2-treated rats at the end of the third week compared to that in model rats (Fig. 2b). IL-6 level was also lower at the end of the second week (*p* < 0.01) in JTW2-treated rats (Fig. 2c). The result suggests that JTW has a beneficial effect on alleviating sleep-loss related inflammation in OR rats.

### The effect of JTW on metabolic parameters in sleep-deprivated OR rats

**Fig. 3:**
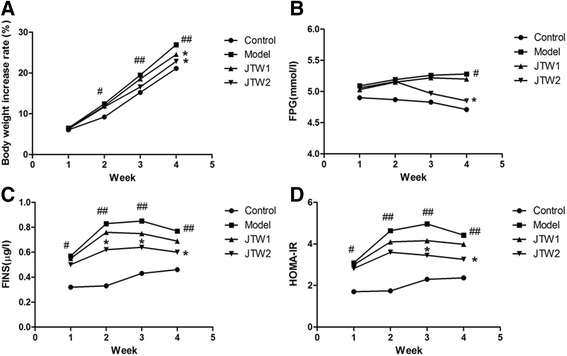
The effect of JTW on metabolic parameters in sleep-deprivated OR rats. JTW treatment decreased the level of metabolic parameters in OR rats with PSD (**a** Body weight increase rate; **b** FPG; **c** FINS; **d** HOMA-IR). ^#^ *p* < 0.05, ^##^ *p* < 0.01, significantly different from control. *p < 0.05, significantly different from model group. Each point represents the mean of 5 rats

The body weight increase rate each week was markedly higher (*p* < 0.01) in model rats than that in control rats (Fig. 3a). It indicates that partially sleep-deprivated OR rats gained more weight during the whole sleep deprivation period. Similar changes were also found in the levels of FINS and HOMA-IR (Fig. 3c-d). However, partial sleep deprivation caused a slight elevation in FPG in model rats and FPG level was significantly higher (*p* < 0.05) than that in control rats at the end of the fourth week (Fig. 3b). Compared with model rats, JTW1 and JTW2-treated rats showed the decreases in body weight increase rate (*p* < 0.05), and JTW2 administration significantly decreased FPG, FINS and HOMA-IR (*p* < 0.05). The differences reached statistical significance at the end of the third or the fourth week (*p* < 0.05) (Fig. 3a-d). The result suggests that chronic sleep deprivation is often associated with insulin resistance which can be attenuated after the intervention of JTW2.

### The effect of JTW on Cry1mRNA,Cry2mRNA and NF-κBmRNA expressions in PBMC of sleep-deprivated OR rats

**Fig. 4:**
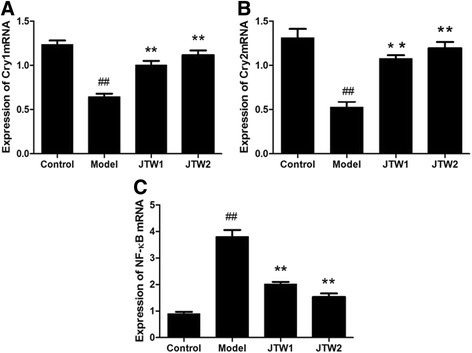
Effects of JTW on Cry1, Cry2, NF-κB mRNA expressions in PBMC of sleep-deprivated OR rats. JTW treatment increased the level of Cry1mRNA, Cry2mRNA expressions and decreased the level of NF-κBmRNA expression in PBMC of sleep-deprivated OR rats (**a** Cry1mRNA; **b** Cry2mRNA; **c** NF-κBmRNA). ^##^ *p* < 0.01, significantly different from control. **p < 0.01, significantly different from model group. Each point represents the mean of 4 rats

As previously reported, clock protein Cry and inflammation mediator NF-κB both regulate the expression of proinflammatory cytokines [28, 29]. The activation of NF-κB signaling pathway is also involved in the development of insulin resistance [30]. Thus we measured Cry1mRNA, Cry2mRNA and NF-κBmRNA expressions in PBMC of sleep-deprivated OR rats. At the end of the fourth week, the Cry1mRNA and Cry2mRNA expressions were markedly decreased while the NF-κBmRNA expression was increased in PBMC of model rats compared with those in control rats (*p* < 0.01) (Fig. 4a-c). However, the administration of JTW1 and JTW2 reversed the above changes followed by PSD (*p* < 0.01) (Fig. 4a-c). The results suggest that the beneficial effects of JTW on inflammation and insulin resistance may be associated with the modulation of circadian clock and inflammation genes expressions in PBMC.

## Discussion

Traditional Chinese medicine has been used to treat insomnia for a long time during Chinese history. However, the underlying mechanism remains unclear. In the present study, we explored the effects and mechanism of JTW on sleep in OR rats with chronic PSD. The result showed that JTW could significantly improve sleep quality in OR rats after sleep curtailment. JTW could also attenuate sleep loss-related inflammation and metabolic disorders. These effects may be associated with the modulation of circadian clock and inflammation genes expressions. All the experimental data indicate that JTW has an obvious hypnotic effect and possesses anti-inflammation effect and insulin sensitizing activity.

Human beings keep wakefulness during the day and sleep at night. This sleep/wake cycle is under the control of circadian clock. Circadian clock is the body’s biological rhythm in keeping with the 24-h day-night cycle or light-dark transitions. In mammals, the circadian timekeeping system is composed of a central clock in the brain’s suprachiasmatic nuclei (SCN) and peripheral clocks in nearly every body cell [31]. At the core of this clock network is a set of interacting transcriptional activators (CLOCK and BMAL1) and repressors (Period and Cryptochrome) [32]. Recent study has found that clock protein Cryptochrome (Cry1 and Cry2) regulates the expression of proinflammatory cytokines. The absence of Cry1 and Cry2 might elevate intracellular cAMP level and activate protein kinase A (PKA), leading to NF-κB activation through phosphorylation of p65 at S276 [28]. NF-κB is one of the most important regulators of various proinflammatory genes expression. Synthesis of inflammatory cytokines, such as TNF-α, IL-1β and IL-6, is all mediated by NF-κB. The activated NF-κB enters the nucleus where it binds to target DNA elements and enhances the transcription of inflammatory genes [29]. This may partially explain the link between circadian rhythm disruption and increased susceptibility to chronic inflammatory diseases.

In the present study, environmental noise seriously disturbed the sleep architecture in OR rat, manifesting as the reduction of total sleep time and total SWS time. SWS plays an important role in glucose regulation and the suppression of SWS may result in decreased insulin sensitivity, leading to impaired glucose tolerance and increased diabetes risk [33]. In addition, environmental noise caused a marked decrease in the expressions of clock gene Cry1mRNA and Cry2mRNA and the increase in the expression of NF-κB mRNA in PBMC, indicating the disruption of the circadian rhythm and the activation of inflammation. As a result, plasma TNF-α, IL-1β and IL-6 levels were increased since these inflammatory cytokines are all secreted from PBMC [34]. However, JTW treatment increased total sleep time and total SWS time in OR rats with PSD while decreased plasma inflammatory cytokines levels. It suggested that JTW has the beneficial effects on sleep loss and the sleep-loss related inflammation. These effects may be associated with the regulation of the expression of Cry1mRNA, Cry2mRNA and NF-κB mRNA.

In addition, chronic PSD by environmental noise led to a significant weight gain and insulin resistance in OR rats, which is consistent with previous report [23]. In Mavanji’s study, the model rats gained more weight than non-deprivated controls after 8 h PSD daily for 9 days. They also found that poor-quality sleep during rest period leads to enhanced food intake and this may contribute to the weight gain in OR rats. Since we did not record the total and daily food intake in the rats, it is lack of evidence to support this idea. This is also one limitation of our study. However, compared with Mavanji’s method, we administered a milder sleep deprivation using a less stressful method (4 h PSD daily for 28 days) to the rats. And we measured the changes of systemic inflammatory condition and metabolic markers which were absent in Mavanji’s study. The result showed that sleep curtailment led to a remarkable increase in body weight, FPG, FINS and HOMA-IR. It may attribute to sleep loss-associated inflammation since there is evidence to suggest that chronic low-grade inflammation is a link between obesity and metabolic syndrome. Many studies have proved that inflammatory cytokines such as TNF-α and IL-6 could decrease insulin sensitivity by negatively affecting the insulin signaling pathway [35, 36]. However, JTW treatment reversed weight gain and improved insulin sensitivity in OR rats with PSD which may be related to its anti-inflammatory effect.

## Conclusions

In conclusion, our study successfully established a rat model of sleep loss, inflammation and insulin resistance. We also demonstrated that JTW improves sleep, inflammation and insulin sensitivity. The therapeutic effect of JTW may mainly be derived from its modulation of circadian clock and inflammation genes expressions in PBMC.

## Abbreviations

Cry: Cryptochrome
EEG: Electroencephalographic
FINS: Fasting insulin
FPG: Fasting plasma glucose
HOMA-IR: HOMA insulin resistance index
HPLC: High performance liquid chromatography
hs-CRP: High-sensitivity C reactive protein
IL-6: Interleukin-6
IR: Insulin resistance
JTW: Jiao-Tai-Wan
NF-κB: Nuclear factor-κB
OR: Obesity-resistant
PBMC: Peripheral blood monocyte cells
PSD: Partial sleep deprivation
SWS: Slow wave sleep
TNF-α: Tumor necrosis factor-α
